# “You Aren’t Alone”: An Analysis of Trans Latinas’ Use of Instagram

**DOI:** 10.3390/ijerph21060699

**Published:** 2024-05-29

**Authors:** Luis R. Alvarez-Hernandez, Kyser Lough, Rebekah Estevez

**Affiliations:** 1 Department of Clinical Practice, School of Social Work, Boston University, 264 Bay State Rd, Boston, MA 02215-1403, USA; 2 College of Journalism & Mass Communication, University of Georgia, 120 Hooper Street, Athens, GA 30602-3018, USA; kyserl@uga.edu; 3 College of Behavioral and Social Sciences, Georgia Southern University, Brannen Hall, 2034, Statesboro, GA 30460-8041, USA; restevez@georgiasouthern.edu

**Keywords:** #translatina, social media, photography, popular culture, hashtags, sense of self, community development, community health

## Abstract

Social media platforms, such as Instagram, provide space for marginalized groups to connect, learn about and express themselves, and cultivate community. Trans Latinas, a group target of violence and discrimination, resist by expressing themselves and building community through social media. As cisgender researchers, we explored how trans Latinas use #translatina on Instagram as a shared space to present themselves and their identities, to leverage this knowledge in our fields. We analyzed 134 posts in February and March of 2020 employing basic and interpretive content analyses while considering Goffman’s theory of presentation of self. Results showed that trans Latinas mostly presented individually through posed selfies taken near the camera, using a straight camera angle, standing, not smiling, and making eye contact. Most users wore makeup, styled hair, and accessories. Analyzing written captions and photos, four themes were constructed to understand how trans Latinas presented their identities and connected with others: (1) expressions of beauty and femininity, (2) fostering community, (3) commercial or work, and (4) feeling good and confident. These results have implications for mental health and health promotion practices, as social media could serve as affirming spaces for trans Latinas to reinforce their self-determination, maintain a sense of self, and build community.

## 1. Introduction

Although research about trans and nonbinary (TNB) communities has increased over the last few decades [1,2], there remains a dearth in the literature regarding the unique experiences of trans Latinas [3]. Trans Latinas are women whose gender identity do not align with their sex assigned at birth, and/or who do not fit the Western notion of binary gender identities [4]. A person who is a trans woman and indicates having ties to a Latin American country can be identified as a trans Latina. Due to oppressive systems like racism and cisnormativity, trans Latinas are at a high risk of experiencing denial and pathologization of identity, job discrimination, intimate partner violence, and housing insecurity [5]. These oppressive experiences can also occur in social media platforms.

Social media spaces can often be a site for violence against women [6], particularly against women of color and who are part of LGBTQ+ communities [7]. For example, LGBTQ+ people experience bullying online even when interacting in virtual spaces they have carved for themselves [8]. These violent experiences often lead users holding marginalized identities, particularly LGBTQ+ individuals and women of color, to balance their online visibility with the negative effects that often follows being visible [9,10]. These users with marginalized identities do not only receive violence in social media spaces from other users, but also from the social media platforms themselves by being censored [11]. However, trans Latinas are also incredibly resilient, cultivating community [12] and naming and defining themselves while engaging in resistance to oppression [13].

Increased use of social media has provided space for marginalized groups to connect with others and learn about and express themselves [14,15]. Research has shown the importance of social media platforms like Instagram and Facebook for marginalized communities to connect, explore, and engage in community organizing and affirmed self-representation [16,17]. Thus, the use of social media is one way that marginalized communities, such as trans Latinas, counter the impact of oppressive contexts that limit identity exploration and development [18]. Further, engaging in social media-based social support can allow trans Latinx (a gender expansive term for Latina/Latino [19]) individuals to see themselves reflected positively in each other, experience affirmation, love, support, and gain access to necessary resources such as shelter and jobs [20].

### 1.1. Social Media and Self-Representation

As previously discussed, authentic self-representation via social media can confer positive mental health experiences for marginalized communities in an accessible way [21,22]. In turn, social media platforms are useful sources of data for understanding self-representation, as these spaces provide insight into the lives of their users [23]. The majority of users engage with social media platforms daily, and the use of Instagram among adults in the United States has risen to 37%, with 51% of Latinx individuals using it [24]. Users’ posts are often about themselves and their friends, documentation of their experiences, social interactions, knowledge of others/surveillance, and escapism [25]. Instagram is additionally highly used by individuals for self-documentation and self-expression as a way of visualizing personalities, personal and social interests, and tastes [25].

Self-portraits, or selfies, are especially seen frequently on Instagram, as users turn the camera toward themselves in acts of self-documentation and self-expression. Selfies offer viewers more than just a visual representation of what the user looks like. As a social practice, they go deeper to show a sense of self-approval and belonging as part of a collective communications practice of self-presentation [26,27]. The high use of Instagram as a mode of self-presentation and self-expression makes Instagram a useful tool for data in the analysis of self-representation [28]. Social media as a space for self-representation offers not only the chance for a user to express themselves but also to express what it means to be a part of their various communities. Through hashtags [29] and imitation [30] in particular, users perform and define their identities and cultivate communities in these online spaces.

### 1.2. Presentation of Self

We apply Goffman’s [31] theory of presentation of self within the social media space to this study. Goffman’s [31] original theory helps to understand in-person social interactions by comparing them to a theater performance, where we construct how others perceive us. Individuals prepare for the performance backstage by attending to appearance (image of the performer), utilizing props (objects to aid the performance), and working as individuals or teams to produce a collective (re)presentation [31]. Applying Goffman’s theory to a technological space, posting to Instagram can be seen as an exhibition and a performance, using the concept of a curator to select and present the content [32]. Additionally, Goffman’s [31] theory of presentation of self, extending from the physical stage into a technological space, means that we are considering multiple socially mediated audiences and subsequent impression management, particularly as a method of defining and reinforcing social norms [33]. Finally, including others in a post using hashtags can invoke the team performance aspect of the presentation of self, and can portray themes of authenticity, family, and community to viewers [34,35]. The original conceptualization included the ability to have immediate criticism from the audience in the form of an interruption, something an online space cannot provide [31]. However, the use of social media as a performance includes the ability for asynchronous interruptive feedback via comments and messages. These interruptions can have both positive and negative effects on the performer, potentially contributing to an unsafe environment online, especially in the case of marginalized populations. Taken together, Goffman’s theory of presentation of self when applied to social media spaces allows us to hold an understanding of the potential purpose of users’ posts regarding self-presentation and community connection.

Importantly, Goffman’s theory has been linked to trans individuals’ use of Facebook, where they may portray an online persona intricate, to their development, community acceptance, and survival [36]. In this study, we work to extend the application of Goffman’s theory to a novel platform—Instagram. For trans Latinas, Instagram becomes a setting where the types of aforementioned performances can take place. For Instagram users, we argue that the front of the performance is a post, which primarily includes the use of images to display the appearance of the users (e.g., makeup, clothing), manners of performance (e.g., physically posing, use of selfies), and the use of props (e.g., picture filters). Other components of an Instagram post, such as captions and the use of hashtags, contribute to the performance of the user and the intended messages for the audience. For trans Latinas, this performance occurs in the context of gender and culture. When applied to social spaces like Instagram, users actively participate as both creators and influencers [30], so these processes happen simultaneously: performance influences gender and culture, and vice versa. Thus, this performance on social media becomes a way that trans Latinas can name, express, and represent themselves.

### 1.3. Current Study

As cisgender scholars with experience in research and practice with trans Latinx communities, trans health, and social media as an expressive visual communication space (see “Researchers’ Positionalities and Reflexivities” below), we comprehend the burden that is often placed on trans community members by cisgender researchers [37]. To ease this burden and consider the heterogeneity of trans experiences [38], social media becomes a space where data are accessible to community outsiders who want a starting place to develop an understanding of trans people’s use of social media. Therefore, we set out to learn from trans Latinas regarding how they utilize #translatina in Instagram as a shared space to present themselves and connect with others who share their identities. Given the multiple sources of data contained in Instagram posts (i.e., pictures, hashtags, captions), the current study was based on two research questions grounded in Goffman’s [31] theory of presentation of self: (1) How do trans Latinas visually present themselves through Instagram posts using the hashtag #translatina? and (2) How is #translatina in Instagram utilized by trans Latinas as a space for connecting with others who share their identities?

## 2. Methods

### 2.1. Data Collection

We selected Instagram posts under #translatina as the source of data. This hashtag was the most used related to trans Latinas, with over 7000 posts from various parts of the world linked to it at the time data were collected. We selected posts from the first two weeks of February 2020 and of March 2020 as our representational random sample “to represent a broad distribution of posting quantity” [23] (p. 117). A total of 74 posts were uploaded from 1 February 2020 to 13 February 2020, while 101 were uploaded from 1 March 2020 to 12 March 2020. Interestingly, while this timeline overlaps with the earliest months of the COVID-19 pandemic, the posts analyzed did not allude to the effects and dynamics that emerged during this time.

**Figure 1:**
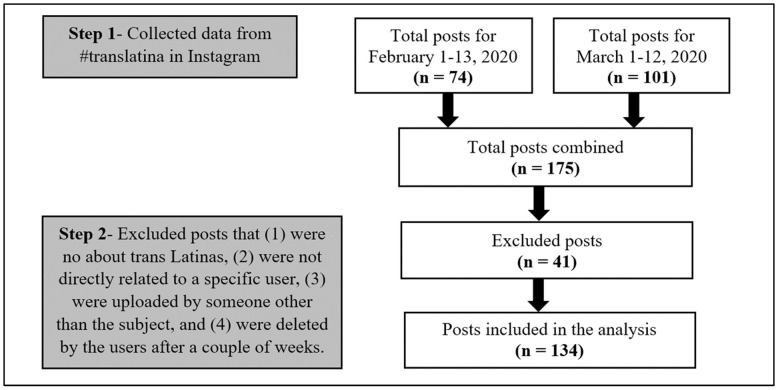
Data collection and exclusion processes.

The focus of this study is on self-representation by trans Latinas; hence, we selected posts where they were visually depicted in still images. The exclusion criteria were: (1) posts that did not have to do with trans Latinas (e.g., language translation services by Latinas), (2) posts not directly related to a specific user (e.g., memes and flyers), (3) posts about people uploaded by someone else and not the user themselves (e.g., photographic series by professional photographers), and (4) posts that were deleted by the users after a couple of weeks (to honor that they no longer wanted their images available to others). Instagram allows users to add more than one image per post; however, we only gathered the first image of the posts that had multiple images. If a post had two pictures in their first image (i.e., a collage), we counted both images as distinct for analysis. If the same user posted similar photos in different posts, they were considered individually as their own post. Of the combined 175 posts, 41 posts were removed, leaving a total of 134 posts that met the inclusion criteria for our analysis (see Figure 1).

Once a final sample was identified, each post (username, picture, caption, and hashtags) was copied and pasted into a slide deck for analysis in their original format, including size, resolution, brightness, etc. Despite these posts being made on publicly accessible Instagram accounts, we felt it ethically important to not include the analyzed images in this manuscript. This decision is due to several factors, including the current hostile sociopolitical climate regarding TNB people in the United States [39] and our desire to avoid potentially publicly outing Latinx TNB community members through publication. We treated the images, captions, usernames, and hashtags culled from Instagram as we would any other form of data (e.g., appropriate secure data storage). No personally identifying information was available, and, therefore, it was not collected nor stored for this study, which also offered privacy for the Instagram users. Importantly, Instagram users’ demographic information (e.g., race, gender identity) are not available for verification. Thus, following methods using Instagram data published elsewhere (see [30]), we relied on demographic descriptions found in the Instagram posts (e.g., #translatina).

### 2.2. Data Analysis

We employed basic content analysis to answer research question 1 and interpretive content analysis to answer research question 2. For research question 1, we created frequencies based on the content of the images, following Drisko and Maschi’s [40] position that basic content analysis is helpful to describe and explain relationships in a dataset. Following best practices for codebook creation, the first and second authors described the first 45 images (34% of the dataset) together and created a codebook to deductively code the rest of the images [40]. The description of the images included the following visual elements:

1. Number of people—Focal subjects in the images as a measure of relationship with others [34,35].
2. Who took the picture?—Evidence that the image was a selfie, channeling Eckel’s [41] broader conceptualization of displayed authorship.
3. Composition—Camera distance and camera angle because of the signified intimacy of a close-up compared to a photo taken further away and the signified power and authority of looking up on the person compared to one looking down on them [42].
4. Clearly posing—Including how they were positioned and how much of the body was visible in the image (this is distinct from camera distance because it focuses on how much of the body is seen versus how far the camera is from the body). We also looked for eye contact and if they were smiling.
5. Appearance—Makeup, styled hair, and use of accessories (e.g., jewelry, glasses, painted nails). Accessories were differentiated from props as being on the person’s body.
6. Filters—Obvious use of software-based filters (e.g., glitter, smoothing of skin).
7. Props—Props were external to the person’s body and intentionally used or emphasized (e.g., mirrors, cellphones).

The first and second author individually coded all pictures using Excel. Both authors then compared codes, redefined coding parameters, and resolved all inter-rater related discrepancies by looking at each image and discussing the new parameters. Most discrepancies occurred due to one coder not being an expert in the technical aspects of photography and the other not being as familiar with trans Latina social experiences but were quickly resolved with subjectivity discussions [43]. For research question 2, we used interpretive content analysis as it allows analysts to describe and create inferential themes regarding latent content like thoughts, emotions, and intentions [40]. This method allowed us to explore how trans Latinas utilized Instagram as a space for connecting with others who share their identities. That is, interpretive content analysis allowed us to deductively code the posts to describe the potential intentions of each user for posting under this specific hashtag. We continued our reflexivity practices throughout the coding process, which involved three steps.

The first and second author initially coded for latent and manifest content in the posts’ pictures, hashtags, and captions using side-by-side coding procedures and reflexive practices to address the “privilege hazard” of our lack of trans lived experiences as cisgender individuals [10] (pp. 153–154). Throughout side-by-side coding, we engaged in steps to ensure “queer data competence”, including being aware of intersectionality and power differences and critically discussing our epistemological assumptions as to the data [10] (pp. 155–157). This step sometimes included searching online for content included in posts to understand their context, such as song lyrics [40].

Next, we developed a codebook with four domains (expressions of beauty and femininity, fostering community, commercial or work, and feeling good and confident). In this phase, all three researchers followed best practices for inter-coder reliability [23] and independently coded the first 30 images (23% of the dataset), achieving 80% agreement by at least two researchers [40]. Team members met and resolved disagreements for the first 30 posts and further defined each theme.

Finally, the rest of the dataset was individually coded by all three researchers using the four themes in the codebook. Inter-reliability was not measured for the entire dataset since the 20% threshold was exceeded when creating the codebook [23]. We discussed coding disagreements and engaged in conversations about differences in our perceptions of codes and posts’ content based on our experiences with trans Latinx communities [43]. Table 1 shows examples of the relationships between codes, themes, and caption text and hashtag data.

### 2.3. Trustworthiness

We engaged in various forms of trustworthiness for both basic content analysis and interpretive content analysis [40]: (1) provided a detailed explanation of the coding processes, (2) maintained a detailed audit trail, (3) engaged continuously in peer debriefing, (4) achieved inter-coder reliability and codebook agreement, and (5) developed a statement of our positionality and reflexivity that guided our interpretation of the data.

#### Researchers’ Positionalities and Reflexivities

Qualitative researchers conducting intersectional research must be reflexive as to how their identities, experiences, and positionalities influence how they analyze data and construct findings [43]. Reflexivity is particularly critical in online research as the researchers navigate insider and outsider positions within the virtual setting [44]. We are also following calls from trans research experts who emphasize the importance of cisgender researchers being transparent with their positionalities when conducting trans-related research [37,45,46]. Hence, all three authors report different positionalities in the context of this study. The first author is a Puerto Rican cisgender gay male. His understanding of trans Latinas’ experiences is based on his social work practice, research, and community engagement with grassroots groups. The first author has engaged in reflexivity about his experiences with privilege and oppression through conversations with the research team, ongoing collaborations with trans Latinas through academic and non-academic activities, and reading and disseminating *testimonios* from the trans Latina community in other research studies. The second author identifies as a white cisgender heterosexual man. As an outsider to both Latinx and trans communities, he understands his lack of direct experience can lead to bias in his interpretations but engaged in regular conversation with the other authors to avoid improper assumptions. The third author identifies as a white cisgender woman with a queer sexual orientation who has strong ties to the community (see [47]). As an insider-outsider [48] to the trans Latinx community, the third author engaged in reflexivity through research team discussion regarding her biases due to her cultural identities and engaged in regular review of the extant literature regarding trans Latinx identities and experiences.

## 3. Results

### 3.1. Descriptive Findings: Visual Presentation

To answer research question 1, Table 2 contains the descriptive frequencies for how trans Latinas present themselves through Instagram posts under #translatina. Overall, of the 134 images, most included only one individual (97.0%), with most images being selfies (64.2%). The images’ composition included users mostly being near the camera (50.7%) while using a straight camera angle (75.4%). Many users seemed to be posing (99.3%), standing (40.3%), not smiling (72.4%), and making eye contact (67.2%). Part of the person’s body could be seen most of the time (63.4%). The location of the users appeared to mostly be inside (76.9%). Most users wore makeup (87.3%), had styled hair (91.0%), and were wearing an accessory (75.4%). Users rarely incorporated a filter (18.7%) or props (22.4%) into their images.

### 3.2. Thematic Findings: #translatina as a Space

Four themes were constructed from the interpretive content analysis to answer research question 2. The themes were (1) expressions of beauty and femininity, (2) fostering community, (3) commercial or work, and (4) feeling good and confident. Translations for captions in Spanish were created by the first author, who is fluent in Spanish, and are included inside brackets.

#### 3.2.1. Expressions of Beauty and Femininity

Posts about expressions of beauty and femininity highlighted traditional feminine aspects of appearance and self-expression. For example, most posts alluded to trans Latina beauty by also including other hashtags (e.g., #webelongtosomethingbeautiful, #transbeauty, #transisbeautiful). Some captions explicitly addressed beauty (e.g., “De qué sirve que tengas ojos bonitos si no ves esta belleza que perdiste frente a ti” [“What is it worth to have pretty eyes if you cannot see this beauty that you lost in front of you”]) and femininity (“Latina barbie doll”).

#### 3.2.2. Fostering Community

Users fostered community through posts that were reflective about relationships, celebrated sisterhood, sought or offered affirmation or validation, and encouraged others to be themselves. Some hashtags that accompanied these posts included #transsisters, #DearTransgirl, #girlslikeus, and #youarenotalone. Many captions were aimed directly at other trans Latina women. For example, many posts under this theme were about the users’ gender transition journey while encouraging readers about their journeys. A user wrote along with pre- and post-transition pictures, “YOU MATTER and your transition is yours to experience […] You aren’t alone. […] YOU GOT THIS. WE GOT THIS”.

#### 3.2.3. Commercial or Work

Users utilized #translatina to promote a product or their services. These products and services highlighted femininity (e.g., makeup, drag performances, and escort services). In some instances, the user tagged or included businesses or products in their posts. Some hashtags in these posts included #maquillaje [makeup], #Escorts, #influencer, and #Working. Some captions included emojis of lips and high heels or music lyrics about physical contact. For instance, a post included Tove Lo’s “Are U gonna tell her?” lyrics, “Our bodies tangled tight in that purple light, we’re making love passed out”.

#### 3.2.4. Feeling Good and Confident

Users posted content where they explicitly shared feeling confident and excited. Users also posted experiences in which they were having a great time and experiencing joy. Some of the hashtags in these posts included #transpower, #bornthisway, #transisbeautiful, and #activist. Users captioned their posts with news about exciting job opportunities, goals they have achieved, their travels, and inspirational quotes. For example, a user captioned their posts, “Feelin cute af today” and “TransLatina Power!!!! Yaaaasssss!”

## 4. Discussion

Emerging research has identified that social media can have a positive impact on mental health for trans community members [49]. As cisgender researchers working in trans Latinx health, advocacy, and visual communication, we sought to explore how trans Latinas visually present themselves through Instagram using the hashtag #translatina and how they may utilize this hashtag as a space for connecting with others who share their identities. We found that trans Latina women used #translatina to visually present themselves in the virtual stage using mostly selfies, with the subject of the photo appearing near to the camera, straight on, standing, making eye contact, and not smiling. The close proximity and eye contact convey a sense of intimacy and visibility to the viewer [42,50], welcoming them in, while the straight-on angle and lack of smile convey a sense of seriousness to their presentation. We see this firm stance as a form of confrontation, where the users resist hiding and instead channel the self-approval and belonging aspects of self-presentation [26,27] to show themselves as both empowered and visible. By purposefully presenting themselves as strong characters in their virtual performances [31], the users expand on the story of trans Latinas as powerful agents of change in our society as told by various traditional written media outlets, such as books that combine trans Latina images with narratives (see [51]). They use the collective norms of Instagram posts, such as selfies and hashtags [26,27,29,30], as a tool for increased representation and recognition, given the need to address online violence [52]. This reinforces how trans Latinas use social media to name and represent themselves, creating and affirming a counternarrative against the backdrop of a society that largely stigmatizes and discriminates against them [5], including their censorship in social media spaces [11].

While part of users’ bodies was mostly seen, they largely utilized indoor settings to take their photographs. Hence, the strong images of trans Latinas were potentially portrayed from inside physical structures like homes and cars for safety. By taking control of their exposure during their performance, users are likely to evade the violence that many trans Latinas experience in society by just existing [5]. The relative physical safety of social media provides users with a sense of control that could be beneficial for their mental health [17].

Similarly, most of the users who posted under #translatina presented a carefully curated self, with makeup, styled hair, and accessories. Appearance [31] is not only important to convey a character’s story, but research has shown that trans individual’s ability to present a gender expression that is congruent with their gender identity is associated with positive mental health outcomes [53]. Of note, most of the women who posted on Instagram under #translatina did not use a filter nor a prop, which are often used to emphasize an additional object or other person as a part of the visual presentation [31]. For those who did utilize a prop, mirrors that reflected the user were common. From a practical standpoint, the mirrors allowed for more of the body to be shown. But, also, this is the user performing from a vulnerable standpoint—looking at themselves in the mirror and presenting both depictions to a viewer brings a sense of self-awareness and intimacy, while inviting the viewer in to see them as they see themselves in the mirror [54]. Vulnerability from the performer and an invitation for empathy by the audience is critical for fostering affirming spaces, including affirming mental health spaces.

Our four themes regarding how women posting under #translatina used Instagram as a space for connecting with others who share their identities add context to these findings. In the first theme, our perceived expressions of beauty and femininity were based on the visual depictions of wearing make-up and having one’s hair styled, as well as photos combined with captions like “Latina barbie doll”. We coded these images as expressions of beauty and femininity because they showed how women posting under #translatina appeared to be striving to fit a certain mold of feminine beauty found in the U.S. Latinx culture. This is aligned with the literature highlighting the ways cisgender and trans Latina women experience pressure to manifest particular standards of beauty. For instance, a recent study by Rojas-Sosa [55] found that a popular beauty magazine targeting Latina women encourages celebration of performances of beauty aligned with white women while regulating aspects that appear “too Latina” in order to regulate and exploit otherness in acceptable ways [55] (p. 55). We see this through examples like hypersexualization in clothing, always taking care to apply make-up and style one’s hair, and taking pride in wearing often painful articles of clothing to always appear in public in certain ways [55]. Thus, the women whose posts fit under this theme appear to be striving to create an appearance aligned with mainstream expectations of Latina femininity [56]. It is also possible that this emphasis and alignment is due to the importance of passing or blending. Passing or blending is often linked to interpersonal safety and experiencing gender affirmation, or the experience of being affirmed and celebrated by others as one’s gender identity through relational experiences, which is often a key component of increasing trans women of color’s wellbeing [57]. Additionally, our definition for expressions of beauty and femininity was linked to users’ appearance, which aligns with previous research showing Latinas’ purposeful cultural and gendered presentation of self in Instagram through a public feminine aesthetic (e.g., big jewelry, long nails) and sexualized body postures [58]. However, due to our limited knowledge of the users’ lives, it is possible that our association between femininity and beauty does not encompass other socially traditional markers of femininity like motherhood, caregiving roles, etc. [59].

In the second theme, fostering community, we describe how users utilized #translatina as a way of connecting not only with Instagram’s audience in general, but with other trans Latinas particularly. Seeing others who reflect one’s similar gender identity and presentation is crucial for trans individuals’ mental health [3], and the users of these posts performed specific roles for other trans Latinas, including sister, confidant, and mentor, through sharing their images and stories in captions. Perhaps most poignantly, there were some women who posted under #translatina who shared images related to their social and/or medical gender transitions with hopeful messages of support for others on their journeys. Trans individuals’ wellbeing and response to discrimination can be mitigated by having mentors [60], so this supportive role of users towards others in the community serves as a hopeful reference for those transitioning or experiencing gender dysphoria. This role of community, be that in person or virtually, cannot be understated for trans people of color, who often use social media to advocate for others in the community [18,61].

In the third theme, commercial or work, we detail how users utilized #translatina as a marketing tool. As many trans Latinas face unemployment and poverty in the United States [5], the users seem to utilize social media as a way to survive and thrive financially. The trans Latinas who used #translatina for commercial or work purposes mostly advertised performances, makeup, and escort services (i.e., #escort). The sexualization of Latinas has often led to their sexual labor, criminalization, and fetishization as ideal objects of desire [62]. The trans Latinas in our sample who engaged in marketing their bodies or image for financial gain, appear to have done so as both the performer and the producer of that performance, potentially enhancing their sense of empowerment. It is also possible that due to the highly stigmatized nature of sex work, women who posted under #translatina who were leveraging Instagram to find work could also be seeking to connect with other women in the industry to cultivate sources of social support and mentorship crucial to their wellbeing.

In the fourth theme, feeling good and confident, we observe how trans Latinas utilized #translatina as a hopeful space to share good news and feelings with others. Social support outside virtual spaces has been essential in the wellbeing of trans Latinas [63]. However, virtual spaces have been vital in the identity development of trans and gender-nonconforming individuals, as they often serve as spaces where people can be their authentic self [64]. It is noteworthy that feeling good and confident is a societal expectation that has been reinforced by social media campaigns like “It Gets Better”, while this expectation of wellness neglects the complex ongoing struggles of trans individuals [65]. By explicitly expressing confidence, affirmation, and gender euphoria in their social media performances [66], the trans Latinas in this study serve as a catalyst for others in the community of the potential of being able to be happy and whole on their own terms.

## 5. Limitations and Contributions to the Literature

As previously discussed, the results of this study were analyzed by cisgender researchers, and only one Latinx-identified researcher, in an effort to decrease the research burden facing trans communities while learning about how trans Latinas may use Instagram to present themselves and connect with others. Thus, while the research team continuously engaged in trustworthiness practices such as reflexivity and engagement with community and literature-based education, there are positionality-based limitations. Additionally, the research team held basic to advanced proficiency in the use of Instagram, and the results are not intended to be generalizable to the use of other social media platforms or to other trans and gender-nonconforming groups (e.g., trans Latino men). There is a probability that many of the posts included in our sample will not be available to researchers in the future, due to removal, deletion and/or privatization of the accounts and posts, whether by the user or the platform, which limits replicability. Additionally, multiple users could be using multiple usernames or accounts at the same time, or different accounts at different points in time. Thus, some of the accounts could also be fake (i.e., posing as someone else), and the images posted by users may not be of themselves. Further, we took at face-value that users posting under #transLatinas identified as trans and Latina. As the study design did not include interviews or other means to contact Instagram users, it is possible that not all posts we analyzed were in fact created by trans Latina women. Finally, given that we collected this study’s data during the earliest months of the COVID-19 pandemic, the posts analyzed did not reference the pandemic. Hence, due to the ever-changing nature of social media platforms and posting trends, future research could analyze posts under #translatina after 2020 and compare the presentation of self of users across time.

Image editing and manipulation is not always apparent. Some of the posts may have been edited in ways that we could not detect, which lends further support to studies that ask users about their Instagram use. The coding of filters in this study, for example, included overlays that added sparkles or extreme changes in overall image tone. A user may have applied a more subtle filter to smooth their skin, and we would not have been able to detect it. However, our study looked from an audience’s perspective, so we were mainly interested in detectable aspects of the construction of the photo.

Although other studies have found social media in general to be partially an affirming space for non-Latinx trans youth [15], our study contributes a useful and richer analysis of digital media as a space of performance for trans Latinas, an under-researched population, by centering their point of view of their identities and performances in our construction of the study results. We also contribute potential methodological implications for future studies that analyze Instagram data with marginalized populations through the application of Goffman’s theory of presentation of self. Moreover, we contribute to the literature the potential ways trans Latinas foster community and create counternarratives to oppression through social media. We began this article by stating that our purpose was to learn from and amplify the voices of trans Latinas regarding how they use social media through an analysis that did not create an initial burden on the community. We hope that this study serves as an example on how to reduce the research burden on trans communities, while also acknowledging the outsider cisgender gaze that shapes our understanding of these communities. Our next steps are to contribute to the literature a deeper understanding of social media use by directly hearing from trans people of color through a qualitative study.

## 6. Implications for Health Promotion and Psychological Practice

Our findings have several implications for health promotion and psychological practice with trans Latinas at the micro-, mezzo-, and macro-levels. At the micro-level, trans Latinas could engage social media as an outlet to become visible and empowered to be their authentic selves in the face of oppression. Mental health practitioners could encourage the use of social media as a space for safety and control that positively contributes to their sense of self and self-determination. At the mezzo-level, trans Latinas could use social media to connect with others in intimate and vulnerable ways. Mental health practitioners, rather than only focusing on referring trans Latinas to clinical synchronous support groups, could aid their clients in using social media to find community, empathy, and affirming communal spaces. From the health promotion perspective, social media could provide spaces where trans Latinas can see others who reflect similar gender identities and presentation and, give hopeful support for transitioning and gender dysphoria. These spaces are critical for trans individuals’ mental health, sense of community, and shared feelings of euphoria. At the macro-level, trans Latinas could use social media for community organizing and advocacy. Mental health and health promotion practitioners could encourage their clients to use social media to learn about organizing efforts and generate change in our society.

## 7. Conclusions

Knowledge regarding the experiences of trans Latinas is scarce in the academic literature [3]. Although social media can project a forced sense of positivity and heteronormativity for trans individuals [65], virtual spaces can serve as mediating stages to build community and to develop and maintain a healthy sense of self. The rising use of social media, including Instagram, offers mental health practitioners, researchers, and activists an opportunity to learn more about this resilient community in potentially less-invasive manners than asking for participant time, energy, and resources in the research process. Our study showed that trans Latinas utilize Instagram to present themselves as strong and beautiful and as sources of support to others, using #translatina. As trans individuals use social media for advocacy [67] and building community [68], we must consider the role of social media in the generation of change and wellbeing within Latinx and TNB communities.

## Figures and Tables

**Table 1:** Examples of the relationships between themes, codes, and data.

Themes	Codes	Caption Text and Hashtag Examples
Theme #1: Expressions of Beauty and Femininity	Posing, inviting us to look at her body, stereotypically feminine body and features.	“Isn’t it lovely?” “La Princesa” “Latina barbie doll” #transbeautiful #transbeauty #transisbeauty
Theme #2: Fostering Community	Encouraging other people in their transition/identity, supportive of others, sharing transition story/journey (vulnerable), connecting with others, referencing and building community (authenticity).	“Grateful for my Trans Family” “It feels amazing to get all the love and support” “You aren’t alone” #deartransgirl #transcommunity #translatinacoalition #girlslikeus #belongtosomethingbeautiful
Theme #3: Commercial or Work	Beauty products placement, use of “shemale” and “travestis,” place, promoting herself (“escorts”), inside a bedroom (more natural and candid and behind the scenes), revealing a seductive outfit and poses.	“Link in bio” Listing and tagging of makeup products and companies. Tagging companies and directly asking them to sponsor her. Using suggestive song lyrics in combination with hashtags. #Shemale #Travestis #Escorts #working
Theme #4: Feeling Good and Confident	Behind the scenes view of her life (not the polished presentation), positive attitudes, smiles, vulnerable and candid and real and authentic and human, process of becoming, normalizing the process.	“Today is going to be a great day” “Knock me down nine times but I get up ten” “Getting closer to my goals” Describing how certain accessories make them feel: “beautiful, worth, confident and fierce AF!” #behappy #positiveday #feelinggorgeous

**Table 2:** Descriptive findings.

Visual Elements	Posts (*N* = 134)
**Number of people or focal subjects** (1 person)	130 (97.0%)
**Who took the picture?**	
Selfie	86 (64.2%)
Not a Selfie	48 (35.8%)
**Composition: Proximity**	
Near	68 (50.7%)
Medium	48 (35.8%)
Far	18 (13.4%)
**Composition: Vantage point**	
Looking up (on the subject)	11 (8.2%)
Looking down (on the subject)	22 (16.4%)
Straight	101 (75.4%)
**Posing** (yes)	133 (99.3%)
**Position**	
Sitting or kneeling	25 (18.7%)
Standing	54 (40.3%)
Leaning	6 (4.5%)
Laying down	8 (6.0%)
Unclear	41 (30.6%)
**Clearly smiling** (yes)	37 (27.6%)
**Eye contact** (yes)	90 (67.2%)
**How much of the person can we see?**	
Part of the body	85 (63.4%)
Most of the body	49 (36.6%)
**Location of the Image**	
Indoors	103 (76.9%)
Outdoors	23 (17.2%)
Unclear	8 (6.0%)
**Appearance**	
Makeup (yes)	117 (87.3%)
Styled hair (yes)	122 (91.0%)
Accessories (yes)	101 (75.4%)
**Filters** (yes)	25 (18.7%)
**Props** (yes)	30 (22.4%)

## Data Availability

No new data were created or analyzed in this study. Data sharing is not applicable to this article.

## References

[1] Moradi B., Tebbe E.A., Brewster M.E., Budge S.L., Lenzen A., Ege E., Schuch E., Arango S., Angelone N., Mender E. (2016). A content analysis of literature on trans people and issues: 2002–2012. Couns. Psychol..

[2] Wanta J.W., Unger C.A. (2017). Review of the transgender literature: Where do we go from here?. Transgender Health.

[3] Abreu R.L., Gonzalez K.A., Mosley D.V., Pulice-Farrow L., Adam A., Duberli F. (2022). “They feel empowered to discriminate against las chicas”: Latina transgender women’s experiences navigating the healthcare system. Int. J. Transgender Health.

[4] Chang S.C., Singh A.A., Dickey L.M. (2018). A Clinician’s Guide to Gender-Affirming Care: Working with Transgender and Gender Nonconforming Clients.

[5] James S.E., Salcedo B. (2017). 2015 U.S. Transgender Survey: Report on the Experiences of Latino/a Respondents.

[6] Donato S., Eslen-Ziya H., Mangone E. (2022). From offline to online violence: New challenges for the contemporary society. Int. J. Sociol..

[7] Dunn S. Technology-Facilitated Gender-Based Violence: An Overview. Centre for International Governance Innovation: Supporting a Safer Internet Paper No 1. https://ssrn.com/abstract=3772042.

[8] Mkhize S., Nunfall R., Gopal N. (2020). An examination of social media as a platform for cyber-violence agaisnt the LGBT+ population. Agenda.

[9] Stegeman H.M., Are C., Poell T. Strategic (in)visibility: How marginalised creators navigate the risks and constraints of online visibility. Proceedings of the the 24th Annual Conference of the Association of Internet Researchers.

[10] Guyan K. (2022). Queer Data: Using Gender, Sex and Sexuality Data for Action.

[11] Are C. (2020). How instagram’s algorithm is censoring women and vulnerable users but helping online abusers. Fem. Media Stud..

[12] Hwahng S.J., Allen B., Zadoretzky C., Barber H., McKnight C., Des Jarlais D. (2019). Alternative kinship structures, resilience and social support among immigrant trans Latinas in the USA. Cult. Health Sex..

[13] Alvarez-Hernandez L.R., Bermúdez J.M. “Me Llamo Luchadora Social”: Trans Latina Immigrants as Nepantleras Generating Change in the Southern U.S.

[14] Lucero L. (2017). Safe spaces in online places: Social media and LGBTQ youth. Multicult. Educ. Rev..

[15] Selkie E., Adkins V., Masters E., Bajpai A., Shumer D. (2020). Transgender adolescents’ uses of social media for social support. J. Adolesc. Health.

[16] Lough K. (2023). Two days, twenty outfits: Coachella attendees’ visual presentation of self and experience on Instagram. J. Vis. Lit..

[17] Cannon Y., Speedlin S., Avera J., Robertson D., Ingram M., Prado A. (2017). Transition, connection, disconnection, and social media: Examining the digital lived experiences of transgender individuals. J. LGBT Issues Couns..

[18] Jackson S.J., Bailey M., Foucault Welles B. (2018). #GirlsLikeUs: Trans advocacy and community building online. New Media Soc..

[19] Vidal-Ortiz S., Martínez J. (2018). Latinx thoughts: Latinidad with an X. Lat. Stud..

[20] Estevez R.I. (2022). “Talk about Resilient-We just don’t Give Up”: The Risk and Resilience Experiences of the Latinx Trans and Nonbinary Community. Doctoral Dissertation.

[21] MacKinnon K.R., Kia H., Lacombe-Duncan A. (2021). Examining TikTok’s potential for community-engaged digital knowledge mobilization with equity-seeking groups. J. Med. Internet Res..

[22] Wright S. (2023). Biohacking queer and trans fertility: Using social media to form communities of knowledge. J. Med. Humanit..

[23] Paulus T.M., Friend Wise A. (2019). Looking for Insight, Transformation, and Learning in Online Talk.

[24] Perrin A., Anderson M. Share of U.S. Adults Using Social Media, Inlcuding Facebook, Is Mostly Unchanged since 2018. https://www.pewresearch.org/fact-tank/2019/04/10/share-of-u-s-adults-using-social-media-including-facebook-is-mostly-unchanged-since-2018.

[25] Lee E., Lee J.A., Moon J.H., Sung Y. (2015). Pictures speak louder than words: Motivations for using Instagram. Cyberpsychol. Behav. Soc. Netw..

[26] Kozinets R., Gretzel U., Dinhopl A. (2017). Self in art/self as art: Museum selfies as identity work. Front. Psychol..

[27] Koliska M., Roberts J. (2015). Selfies: Witnessing and participatory journalism with a point of view. Int. J. Commun..

[28] Maddox J. (2021). “Be a badass with a good ass”: Race, freakery, and postfeminism in the #StrongIsTheNewSkinny beauty myth. Fem. Media Stud..

[29] Matley D. (2017). ‘This is NOT a #humblebrag, this is just a #brag’: The pragmatics of self-praise, hashtags and politeness in Instagram posts. Discourse Context Media.

[30] Mosley D.V., Abreu R.L., Ruderman A., Crowell C. (2017). Hashtags and hip-hop: Exploring the online performances of hip-hop identified youth using Instagram. Fem. Media Stud..

[31] Goffman E. (1959). The Presentation of Self in Everyday Life.

[32] Hogan B. (2010). The presentation of self in the age of social media: Distinguishing performances and exhibitions online. Bull. Sci. Technol. Soc..

[33] Baym N.K., Boyd D. (2012). Socially mediated publicness: An introduction. J. Broadcast. Electron. Media.

[34] Lui R., Suh A. (2017). Self-branding on social media: An analysis of style bloggers on Instagram. Procedia Comput. Sci..

[35] Smith L.R., Sanderson J. (2015). I’m going to Instagram it! An analysis of athlete self-presentation on Instagram. J. Broadcast. Electron. Media.

[36] Gadgil G., Prybutok G., Prybutok V. (2023). Mediation of transgender impression management between transgender privacy paradox and trans Facebook persona: A trans perspective. Comput. Human. Behav..

[37] Vincent B.W. (2018). Studying trans: Recommendations for ethical recruitment and collaboration with transgender participants in academic research. Psychol. Sex..

[38] Tebbe E.A., Budge S.L. (2016). Research with trans communities: Applying a process-oriented approach to methodological considerations and research recommendations. Couns. Psychol..

[39] Dhanani L.Y., Totton R.R. (2023). Have you heard the news? The effects of exposure to news about recent transgender legislation on transgender youth and young adults. Sex. Res. Soc. Policy.

[40] Drisko J.W., Maschi T. (2016). Content Analysis.

[41] Eckel J., Eckel J., Ruchatz J., Wirth S. (2018). Selfies and authorship: On the displayed authorship and the author function of the selfie. Exploring the Selfie: Historical, Theoretical, and Analytical Approaches to Digital Self-Photography.

[42] Berger A.A., Adler R.P. (1981). Semiotics and TV. Understanding Television: Essays on Television as a Social and Cultural Force.

[43] Esposito J., Evans-Winters V. (2022). Introduction to Intersectional Qualitative Research.

[44] Salmons J.E. (2022). Doing Qualitative Research Online.

[45] Restar A.J. (2023). Why confronting positionality matters to advancing trans research and discourse. Lancet Reg. Health Am..

[46] Veale J.F., Deutsch M.B., Devor A.H., Kuper L.E., Motmans J., Radix A.E., Amand C.S. (2022). Setting a research agenda in trans health: An expert assessment of priorities and issues by trans and nonbinary researchers. Int. J. Transgender Health.

[47] Estevez R., Merrifield J., Delgado-Romero E.A., Sena A.E. (2023). Dichos for culturally responsive practice: LGBTQ+ Latinxs. Latinx Mental Health: From Surviving to Thriving.

[48] Dwyer S.C., Buckle J.L. (2009). The space between: On being an insider-outsider in qualitative research. Int. J. Qual. Methods.

[49] Simms D. (2020). Peer responses to trans youth tweeting about self-harm and suicidality. Creat. Nurs..

[50] Coleman R., Kuypers J.A., D’Angelo P. (2010). Framing the pictures in our heads. Doing News Framing Analysis: Empirical and Theoretical Perspectives.

[51] Delgado Lopera J. (2014). ¡Cuéntamelo! Oral Histories by LGBT Latino Immigrants.

[52] Colliver B. (2023). Responding to Transphobic Violence Online.

[53] Costa R., Colizzi M. (2016). The effect of cross-sex hormonal treatment on gender dysphoria individuals’ mental health: A systematic review. Neuropsychiatr. Dis. Treat..

[54] Lutz C., Collins J. (1991). The photograph as an intersection of gazes: The example of National Geographic. Vis. Anthropol. Rev..

[55] Rojas-Sosa D. (2020). ‘Should Latinas go blond?’ Media representation and the regulation of Latina bodies and Latinas’ social and cultural practices in a beauty magazine. Gend. Lang..

[56] Molina-Guzman I. (2010). Dangerous Curves: Latina Bodies in the Media.

[57] Sevelius J.M. (2013). Gender affirmation: A framework for conceptualizing risk behavior among transgender women of color. Sex. Roles.

[58] Barreto A. (2018). Ni Santas Ni Putas, Sólo Mujeres: Disrupting Appropriate Latina Femininity through Raunch Aesthetics on Instagram. Master’s Thesis.

[59] Alvarez-Hernandez L.R., Bermúdez J.M. (2023). Entre madres y comadres: Trans Latina immigrants empowering women beyond marianismo. Affilia.

[60] Truszczynski N.A. (2019). The Coping Strategies and Resilience of Trans and Non-Binary People in Response to Identity-Based Discrimination. Ph.D. Thesis.

[61] Fischer M. (2016). #Free_CeCe: The material convergence of social media activism. Fem. Media Stud..

[62] Rodríguez J.M. (2023). Puta Life: Seeing Latinas, Working Sex.

[63] Rhodes S.D., Alonzo J., Mann L., M Simán F., Garcia M., Abraham C., Sun C.J. (2015). Using photovoice, Latina transgender women identify priorities in a new immigrant-destination state. Int. J. Transgenderism.

[64] Raun T. (2015). Video blogging as a vehicle of transformation: Exploring the intersection between trans identity and information technology. Int. J. Cult. Stud..

[65] Haimson O.L. (2020). Challenging “getting better” social media narratives with intersectional transgender lived experiences. Soc. Media Soc..

[66] Haimson O.L., Dame-Griff A., Capello E., Richter Z. (2021). Tumblr was a trans technology: The meaning, importance, history, and future of trans technologies. Fem. Media Stud..

[67] Williams M.L., Langmia K., Tyree T.C.M. (2017). “I don’t belong in here!” A social media analysis of digital protects, transgender rights, and international restroom legislation. Social Media: Culture and Identity.

[68] Stone A.L., Nimmons E.A., Salcido R., Schnarrs P.W. (2020). Multiplicity, race, and resilience: Transgender and non-binary people building community. Sociol. Inq..

